# Comments on “Bicarbonate and Serum Lab Markers as Predictors of Mortality in the Trauma Patient”

**DOI:** 10.5811/westjem.31021

**Published:** 2024-09-25

**Authors:** Patrick McGinnis, Samantha Camp, Minahil Cheema, Shriya Jaddu, Quincy Tran, Jessica Downing

**Affiliations:** * University of Maryland School of Medicine, Baltimore, Maryland; † University of Maryland Medical Center, Baltimore, Maryland

Dear Editor:

We read with great enthusiasm the article “Bicarbonate and Serum Lab Markers as Predictors of Mortality in the Trauma Patient” by Talbott et al,^1^ which was recently published in the *Western Journal of Emergency Medicine*. Talbott et al utilized the TriNetX database to explore associations between serum laboratory markers (bicarbonate, lactate, and base excess) and 30-day mortality. Despite the novelty of their findings, we have a few comments about the methodology and results that may not support their conclusions.

Our first comment is with regard to the lack of stratification of anatomical location and trauma type. While it is commendable to employ large sample sizes to improve generalizability, it is less helpful for clinicians who manage various types of traumas. For instance, patients who sustain gunshot wounds to the thigh may suffer hemorrhagic shock and metabolic acidosis producing elevated lactate values,^2^ yet they have a higher chance of survival if resuscitation is prompt.^3^ Several studies have shown lactate levels from patients with blunt traumatic brain injury are indistinguishable from healthy controls, yet these patients’ risk of mortality is increased due to the location of injury.^4^ These patients activate different treatment algorithms, making conclusions about “trauma” patients too broad for clinical utility.

Moreover, the methodology for patient selection could be more narrowly defined. The authors used the International Classification of Diseases, 10^th^ Revision, Clinical Modification (ICD) code ICD 10 CM: T14 Injury to any unspecified body region, which includes any injury from a superficial injury to traumatic amputation. This poses a wide spectrum of treatments and survivability, as injury location impacts the triage process and mobilization of staff and resources.

We replicated Talbott’s analysis with a narrower anatomical location using ICD 10 CM: S06 Intracranial injury including subarachnoid and subdural hemorrhage, both of which raise suspicion for elevated lactate. We agree higher lactate values are associated with increased risk of mortality; however, specifying the location of injury (intracranial) strengthened this association beyond the Talbott et al analysis. After stratifying lactate by increments of 2 we found that the risk of mortality was 1.06%, 3.42%. 13.3%, 27.4%, 38.1%, and 57.0% for lactate ranges of <2, 2–4, 4–6, 6–8, 8–10, and 10–12 millimoles per liter, respectively. These results illustrate how specifying an injury type and anatomical location provides narrower, more useful associations between serum lab markers and 30-day mortality.

Finally, Talbott et al used “same day” lactate levels. Although lactate at arrival may have prognostic value, persistently elevated level after 20 hours of injury portends different prognoses.^5^ When providing guidance to clinicians treating trauma patients, this granularity aids in understanding a patient’s clinical condition, as outcomes for patients with elevated arrival lactate that is cleared after four hours vs those with persistently elevated lactate at 24 hours are vastly different.^6^ Nonetheless, we commend the work of Talbott et al and are excited about the magnitude of this study. We recommend that clinicians be prudent in interpreting the study’s result and in applying them to their clinical practice.
